# Forecasting Malaria Morbidity to 2036 Based on Geo-Climatic Factors in the Democratic Republic of Congo

**DOI:** 10.3390/ijerph191912271

**Published:** 2022-09-27

**Authors:** Eric Kalunda Panzi, Ngianga II Kandala, Emery Luzolo Kafinga, Bertin Mbenga Tampwo, Ngianga-Bakwin Kandala

**Affiliations:** 1 Département de la Santé Communautaire, Institut Supérieur des Techniques Médicales de Kinshasa (ISTM/Kin), Kinshasa B.P. 774, Congo; 2 School of Health and Care Professionals, Faculty of Science and Health, University of Portsmouth, Portsmouth PO1 2QG, UK; 3 Department of Epidemiology and Biostatistics, Schulich School of Medicine and Dentristy, Western University, London, ON N6G 2M1, Canada; 4 Division of Epidemiology and Biostatistics, School of Public Health, University of the Witwatersrand, Johannesburg 2193, South Africa; 5 Warwick Medical School, University of Warwick, Coventry CV4 7AL, UK

**Keywords:** forecasting, model, malaria morbidity, geo-climatic factors, DRC

## Abstract

Background: Malaria is a global burden in terms of morbidity and mortality. In the Democratic Republic of Congo, malaria prevalence is increasing due to strong climatic variations. Reductions in malaria morbidity and mortality, the fight against climate change, good health and well-being constitute key development aims as set by the United Nations Sustainable Development Goals (SDGs). This study aims to predict malaria morbidity to 2036 in relation to climate variations between 2001 and 2019, which may serve as a basis to develop an early warning system that integrates monitoring of rainfall and temperature trends and early detection of anomalies in weather patterns. Methods: Meteorological data were collected at the Mettelsat and the database of the Epidemiological Surveillance Directorate including all malaria cases registered in the surveillance system based on positive blood test results, either by microscopy or by a rapid diagnostic test for malaria, was used to estimate malaria morbidity and mortality by province of the DRC from 2001 to 2019. Malaria prevalence and mortality rates by year and province using direct standardization and mean annual percentage change were calculated using DRC mid-year populations. Time series combining several predictive models were used to forecast malaria epidemic episodes to 2036. Finally, the impact of climatic factors on malaria morbidity was modeled using multivariate time series analysis. Results: The geographical distribution of malaria prevalence from 2001 and 2019 shows strong disparities between provinces with the highest of 7700 cases per 100,000 people at risk for South Kivu. In the northwest, malaria prevalence ranges from 4980 to 7700 cases per 100,000 people at risk. Malaria has been most deadly in Sankuru with a case-fatality rate of 0.526%, followed by Kasai (0.430%), Kwango (0.415%), Bas-Uélé, (0.366%) and Kwilu (0.346%), respectively. However, the stochastic trend model predicts an average annual increase of 6024.07 malaria cases per facility with exponential growth in epidemic waves over the next 200 months of the study. This represents an increase of 99.2%. There was overwhelming evidence of associations between geographic location (western, central and northeastern region of the country), total evaporation under shelter, maximum daily temperature at two meters altitude and malaria morbidity (*p* < 0.0001). Conclusions: The stochastic trends in our time series observed in this study suggest an exponential increase in epidemic waves over the next 200 months of the study. The increase in new malaria cases is statistically related to population density, average number of rainy days, average wind speed, and unstable and intermediate epidemiological facies. Therefore, the results of this research should provide relevant information for the Congolese government to respond to malaria in real time by setting up a warning system integrating the monitoring of rainfall and temperature trends and early detection of anomalies in weather patterns.

## 1. Introduction

The fight against climate change, good health and well-being are, respectively, the thirteenth and the third Sustainable Development Goals (SDGs) of the United Nations. Section 3.3. of the third SDG aims to end the HIV/AIDS epidemic, tuberculosis, malaria and neglected tropical diseases and to combat hepatitis, water-borne diseases and other communicable diseases by 2030.

Global warming is the phenomenon of increasing average ocean and air temperatures, induced by the quantity of heat trapped on the earth’s surface. In humanitarian terms, an increase in extreme weather events leads to torrential rains, storms, droughts, as well as an increase in diseases with epidemic potential such as malaria, diarrheal and respiratory diseases.

Malaria is a vector-borne disease whose existence and transmission depend on three main factors: the Plasmodium parasite, the Anopheles vector and the human host. Beyond these essential factors, the risk of malaria transmission can be maintained or increased by environmental or climatic conditions as well as socio-economic factors [1].

In the past, several authors had already highlighted the relationship between climate variations and malaria endemicity [2,3,4].

Malaria constitutes a global burden in terms of morbidity and mortality. In 2019, 229 million malaria cases in 91 countries were recorded, an increase from 211 million cases in 2015. Globally, the number of deaths from malaria was estimated at 409,000 in 2019, compared with 446,000 in 2015. The WHO African Region carries a disproportionately high share of the global malaria burden with 94% of malaria cases and deaths occurring in 2019 [5].

Environmentally related morbidity and mortality still remain high globally, although they have declined significantly in recent decades [6].

In sub-Saharan Africa in general and in the Democratic Republic of Congo (DRC) in particular, where malaria represents the greatest burden of disease, studies on forecasting malaria prevalence based on an environmental and longitudinal approach are rare.

Much published research on the relationship between malaria and climate change has emphasized on the increased risk posed by the expansion of malaria-prone areas. In some parts of Africa, the number of malaria cases is decreasing and scientists believe that this is due not only to prevention measures but also to the effects of climate change on rainfall [7,8,9].

The purpose of this study is to forecast malaria episodes to 2036 using time series forecasting models in order to strengthen the surveillance and response to diseases with epidemic potential throughout the DRC.

## 2. Materials and Methods

### 2.1. Materials

#### 2.1.1. Study Areas

**Figure 1:**
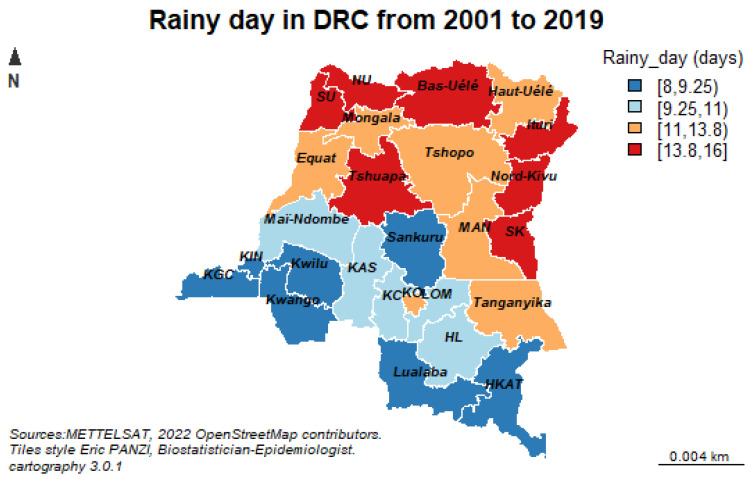
Map of the DRC showing the 26 administrative provinces with average number of YEARLY rainy days between 2001 and 2019 obtained from the National Geographic Institute of the DRC.

This study was conducted in the Democratic Republic of Congo (DRC), which has 26 administrative provinces (See Figure 1). The DRC is located in the heart of Africa and is one of the largest countries on the continent with an area of 2,345,000 km^2^. It shares 9165 km of borders with nine neighboring countries: the DRC to the west, Uganda, Burundi, Rwanda and Tanzania to the east, the Central African Republic and Sudan to the north and Zambia and Angola to the south. The country’s extensive borders, combined with a lack of transport and communication infrastructure, make it particularly difficult to trade and move goods and people within the country.

The United Nations World Population Prospects estimates the 2022population of DRC to be 95,240,792 inhabitants with a population growth rate of 3.19%, the highest in the world and the population projected to surpass 100 million in 2024 and double its population by 2047. The fertility rates in DRC of 6.11 births per woman is also among the highest in the world. However, since the invasion of DRC in 1996 by neighbouring countries mainly Rwanda and Uganda and the second war of invasion in 1998, also called the African world war, the deadliest conflict on earth since the Chinese Civil War with an estimated over 6 million civilian deaths from 1993 to 2003 alone and 47% of the deaths were children under 5. In the economic war in DRC, which is still ongoing, 90% of soldiers died from malaria and disease and displaced a huge percentage of the population, which makes the DRC one of the most challenging environments for health development in Africa with many issues that a rapidly increasing population can exacerbate such as ongoing conflict, lack of modern health services in addition to major health problems, the EBOLA pandemic, HIV/AIDs as well as rape, human trafficking and child labor [10,11].

#### 2.1.2. Type of Study and Data Collection

This was a retrospective longitudinal ecological study. We exploited the Mettelsat database for meteorological data on the one hand and the Epidemiological Surveillance Directorate (ESD) database including malaria data from 2001 to 2019 on the other hand.

The ESD database is centralized data of 26 provincial health directorates of the DRC including all malaria cases registered in the surveillance system based on positive blood test results, either by microscopy or by a rapid diagnostic test for malaria, which was used to estimate malaria morbidity by province of the DRC.

**Figure 2:**
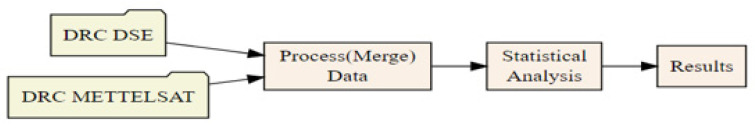
Data collection flow chart. DRC METTELSAT; National Agency of Meteorology and Remote Sensing by Satellite of the DRC. DRC DSE: Epidemiological Surveillance Directorate of the DRC.

Climatic data, including total rainfall quantity, temperature, wind speed, total evaporation under shelter, and average relative humidity, were measured on a permanent basis using different weather stations in the DRC. The distribution of these stations takes into account the variability of the different eco-zones with respect to their climate, their proximity to rivers and altitude. Geographic data are collected at the National Geographic Institute including longitude, latitude, altitude, seasons and hydrography (Figure 2).

This figure shows that malaria and climate data were collected separately and then merged for the analyses.

### 2.2. Sampling

**Figure 3:**
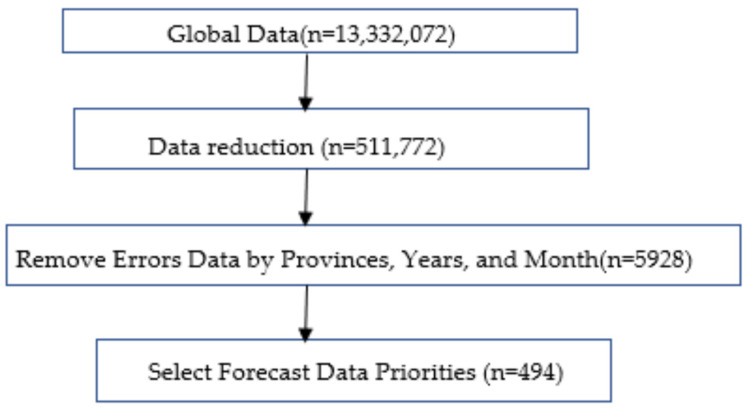
Sample flow chart for the analysis.

Sampling was conducted in three stages as follows: In the first stage, we drew lots from the Provincial Health Directorates (PHD) with at least one weather station. In total, the DRC has 26 PHD. For the malaria data, 13,332,072 observations were selected for our analysis. These are 26 provinces × 19 years × 52 epidemiological weeks × 519 Health Zones or the total of 13,332,072. In the second stage, we selected the health zones with meteorological data. We reduced the observations by grouping them into 26 provinces, 19 years, 52 epidemiological weeks. Outliers and incomplete data reported by some health zones represented less than one percent for the 26 provinces from 2001 to 2019 (Figure 3). Outliers were removed during the data cleaning process. Missing data were replaced by the mean value imputation technique. In the third stage, we have the malaria cases completely and correctly recorded in the database. The data were reduced in terms of 26 provinces and 19 years. This gives a total of 494. This allowed us to forecast malaria prevalence to 2036 (Figure 3).

#### Statistical Methods

The raw data from the different sources mentioned above were cleaned, recoded and merged into a single Excel file. The imputation technique of mean values was used to fill in missing data. The data were analyzed using Stata, R for Windows 4.1.3, R-studio 2022.02.3 (Vienna, Austria) and ARC-GIS 10.2 (ESRI, Redlands, CA, USA). Flat sorting and calculations of measures of association were performed on the collected data. Variables were compared using Spearman correlation analysis. Finally, the modeling and forecasting of malaria epidemic episodes to 2036 was done by combining several time series models as follows:

Let $X_{t}\;$ be a time series with the following general additive structure:

$$X_{t}\;=m_{t}+s_{t}+\varepsilon_{t}$$

$m_{t}$ is the trend which can be linear, polynomial, exponential… It corresponds to the evolution over time independently of seasonal fluctuations.

$s_{t}$ is the seasonality, i.e., a periodic component of period d such that $\exists d,\forall t,s_{t+d}+s_{t}$. Seasonality corresponds to pure seasonal variations. $\varepsilon_{t}$ is the residual or noise of the time series.

The trend and seasonality correspond to the deterministic part of the series, the noise is the random component.

The study of time series has two aspects, on the one hand the extraction of the deterministic character and on the other hand the determination of a model for the noise. These two steps are necessary to be able to make forecasts.

**Figure 4:**
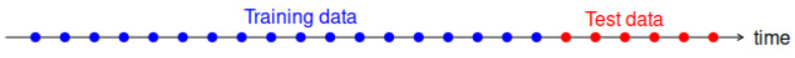
Training set and a test set.

To evaluate the predictive performance of a model, it is common to divide the available data into a “training set” and a “test set”, i.e., to separate the data set into two parts: one to estimate the number and value of the model’s parameters, the other to test the model’s predictions [10] (Figure 4). The use of a test set allows us to know if the model makes relevant predictions when confronted with new data. In this case, we have divided our data series into a training set and a test set of sizes 90% (or 205 months) and 10% (or 23 months), respectively. Thus, we calculated the forecast errors on a test data set size of 228 months (19 years at a frequency of 12 months). Sequential ensemble methods were used to select the training set and the test set.

The most commonly used forecast validation metrics are the MAE (Mean Absolute Error) and the RMSE (Root Mean Squared Error) whose formulas are as follows:

$$RMSE=\sqrt{\frac{1}{n}\overset{N}{\underset{i=1}{\sum }}{\left(\hat{y}i-yi\right)}^{2}}$$

$$MAE=\mathrm{mean}\left(\left|e_{t}\;\right|\;\right)$$

With:

$$e_{t}=error=\;\hat{y}i-yi|i-1\;$$

$$MAPE=\frac{1}{n}\;\frac{\sum_{i=1}^{N}\left|\;\hat{y}i-yi\;\right|\;}{\left|yi\right|}$$

with:

*n* = number of data
ŷ*i* = predicted data
*yi* = reserved data

Optimal model selection [12,13].

The Box-Jenkins SARIMA approach and the combination of several forecasting models (ETS, ARIMA, STL-ETS, NNAR, and TBATS) was implemented. Using malaria case count data from the last 19 years (228 months) of observations, we model and forecast malaria episodes to 2036.

## 3. Results

### 3.1. Spatial and Temporal Distribution of Malaria Morbidity and Mortality

**Figure 5:**
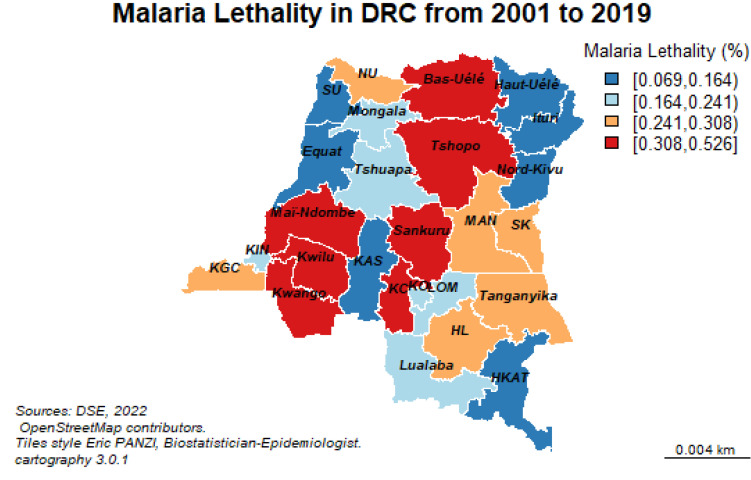

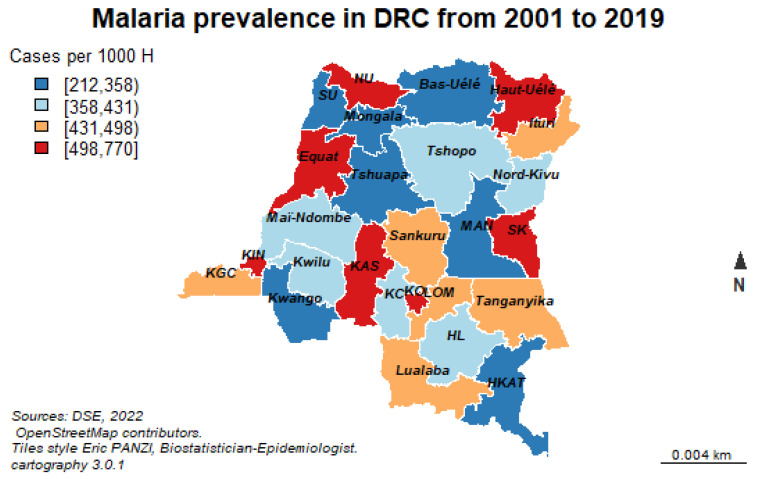
Spatial distribution of malaria morbidity and mortality from 2001 to 2019. Note: The legend of the provinces, see the Appendix A and Appendix B.

Malaria prevalence in South Kivu was 7700 cases per 100,000 people at risk over the past 19 years. Several provinces reported between 4980 and 7700 cases per 100,000 people exposed. In the northwestern part, there is Kinshasa, Equateur and North-Ubangi. In the center of the country, we find Kasai Central and Oriental. In the southeastern part of the country, there is Haut-Uélé and Sud-Kivu. The orange color symbolizes the malaria prevalence ranging from 4310 to 4980 cases per 100,000 exposed persons (Figure 5).

Malaria killed the most people in Sankuru with a case fatality rate of 0.526%, followed by Kasai (0.430%), Kwango (0.415%), Bas-Uélé (0.366%) and Kwilu (0.346%).

### 3.2. Temperatures (in °C)

**Figure 6:**
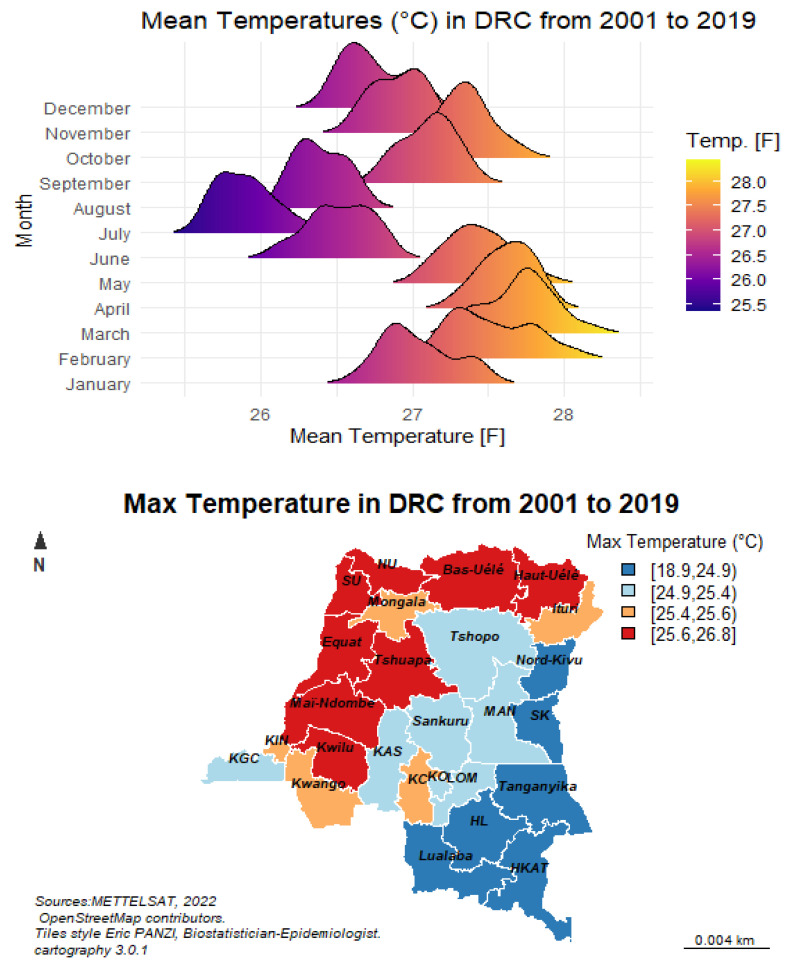
Evolution of average temperatures from 2001 to 2019 (for the legend of the provinces, see the Appendix A and Appendix B).

Describing the variability of temperatures, it is very useful to carry out a detailed analysis of the results obtained with these variables. In this case, it is necessary to determine the trends in temperature values over the 19-year period from 2001 to 2019 (Average temperatures are calculated by year and by province). The three variables in question (average temperatures [Tmean], maximum temperatures [Tmax] (Figure 6) and minimum temperatures [Tmin]) therefore take into account the air temperature values near the surface, and are presented separately. The following figure describes the variability of near-surface air temperature for the different measurement stations considered.

**Figure A1:**
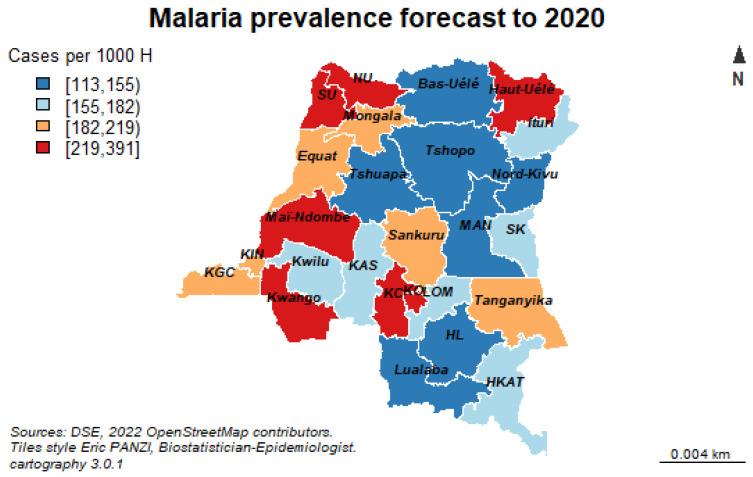
Malaria prevalence forecasts to 2020.

**Figure A2:**
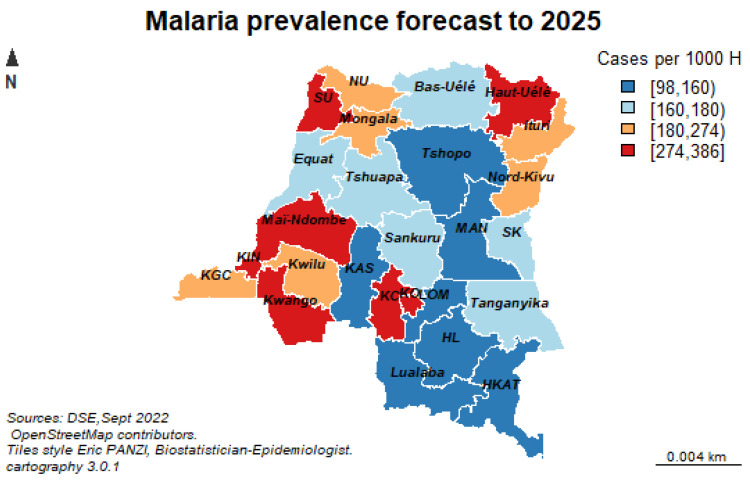
Malaria prevalence forecasts to 2025.

**Figure A3:**
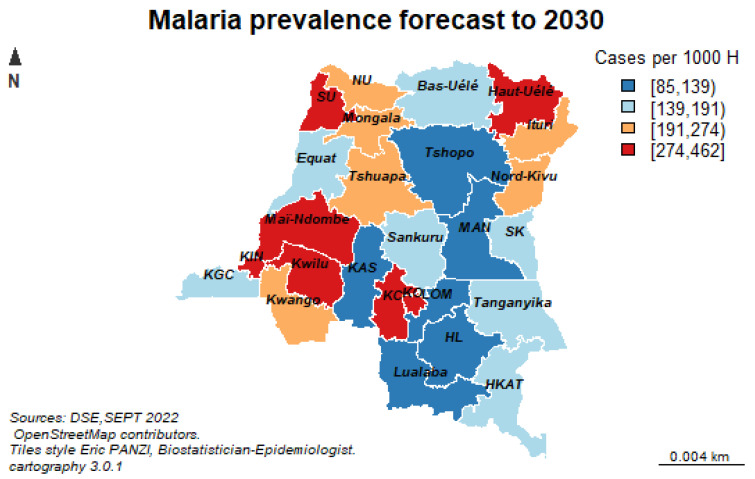
Malaria prevalence forecasts to 2030.

**Figure A4:**
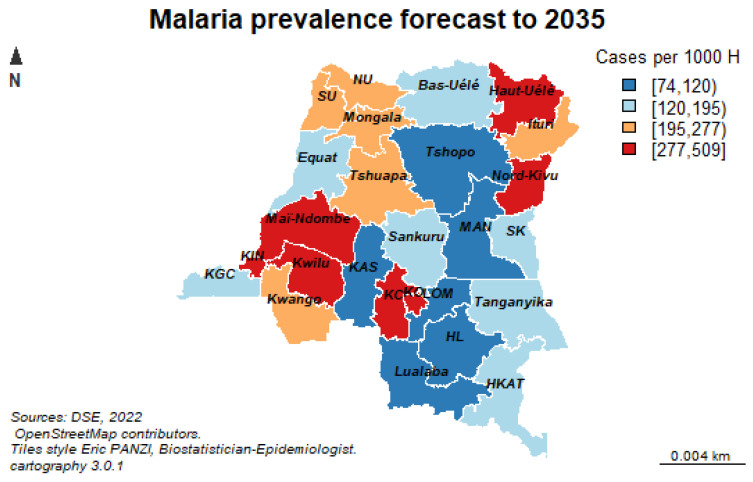
Malaria prevalence forecasts to 2035.

**Figure A5:**
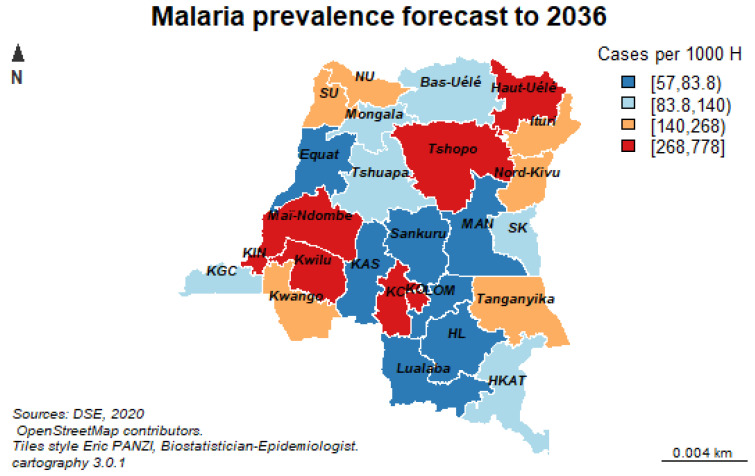
Malaria prevalence forecasts to 2036.

**Figure A6:**
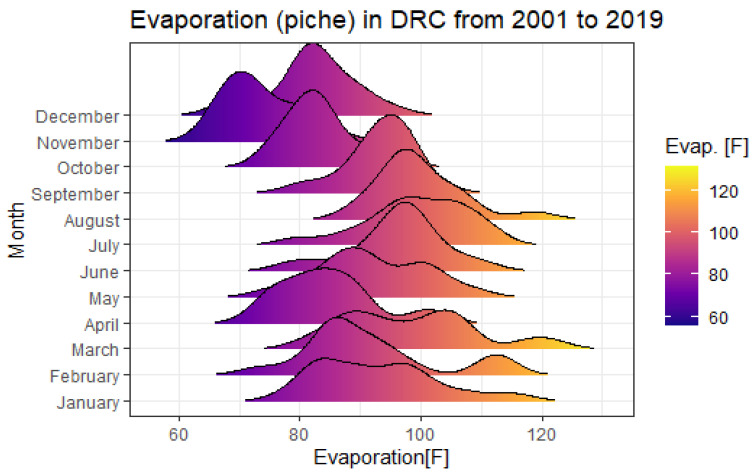
Evaporation (Piche) in DRC from 2001 to 2019.

**Figure A7:**
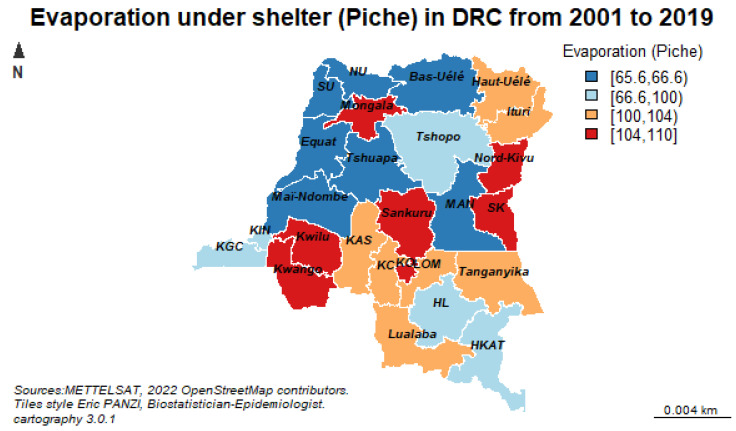
Total evaporation under shelter (Piche) in DRC from 2001 to 2019.

**Figure A8:**
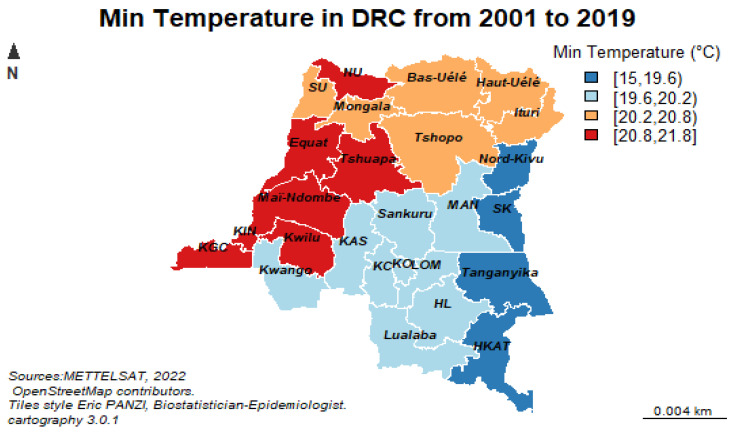
Min temperature (°C) in DRC from 2001 to 2019.

**Figure A9:**
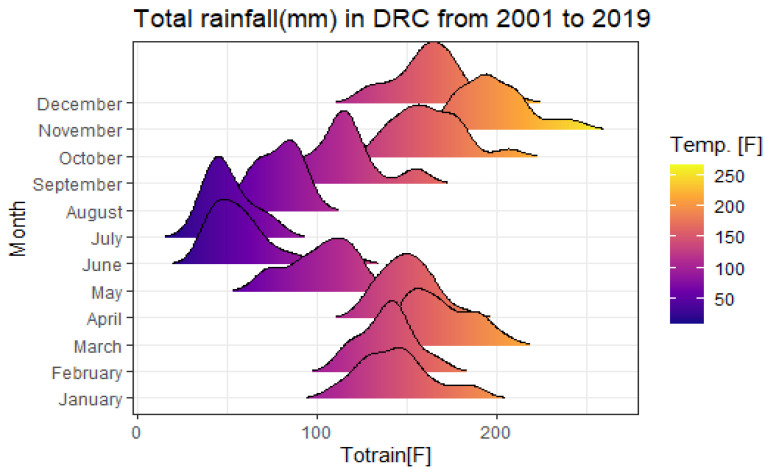
Total rainfall (mm) in DRC from 2001 to 2019.

**Figure A10:**
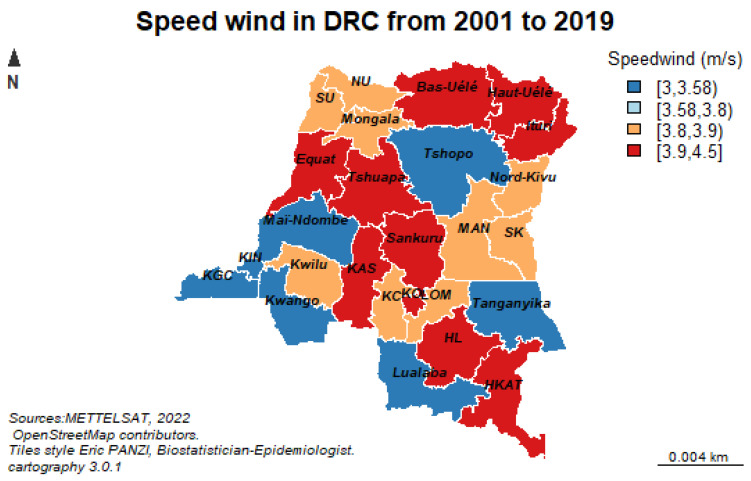
Speed wind (m/s) in DRC from 2001 to 2019.

**Figure A11:**
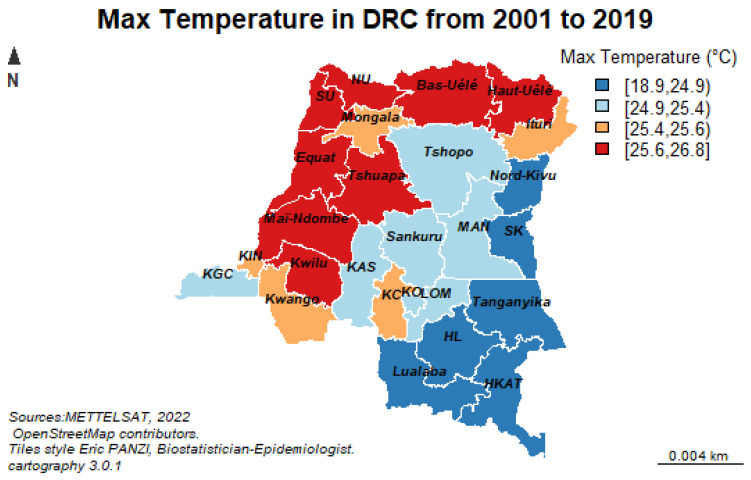
Max temperature (°C) in DRC from 2001 to 2019.

**Figure A12:**
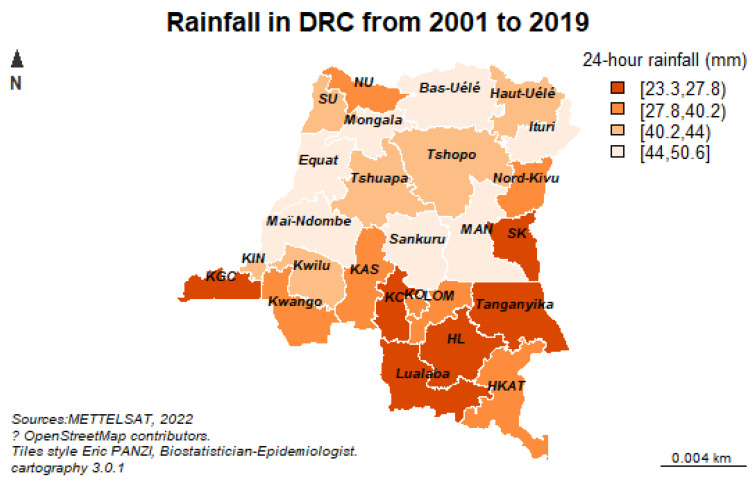
Rainfall (mm) in DRC from 2001 to 2019.

Distributed by geographical entities, it is clear that the average temperatures are divided into three regions. The northwestern part is the warmest with average temperatures ranging from 25.4 °C to 26.8 °C. In Tshopo and the center of the DRC, temperatures range from 24.9 to 25.6 °C. Finally, it is between 18.9 and 24.9 °C in the southeastern part of the country. Over the course of the year, temperatures begin to rise gradually from January to May. These increases are between about 1 °C and 2.7 °C. Then, a significant drop is seen between June and August. This cycle starts again from September to January of the next year. Other maps showing weather variables and malaria forecast maps can be found in the Appendix A and Appendix B (Figure A1, Figure A2, Figure A3, Figure A4, Figure A5, Figure A6, Figure A7, Figure A8, Figure A9, Figure A10, Figure A11 and Figure A12).

### 3.3. Time Series Profile

The different steps of the stochastic modeling of the Malaria series are the following: (1) Identification of the series, (2) Diagnosis of the series, (3) Choice of model, (4) Diagnosis of the quality of the model, (5) Adjustment of the model, (6) Validation of the model, (7) Forecasting.

**Figure 7:**
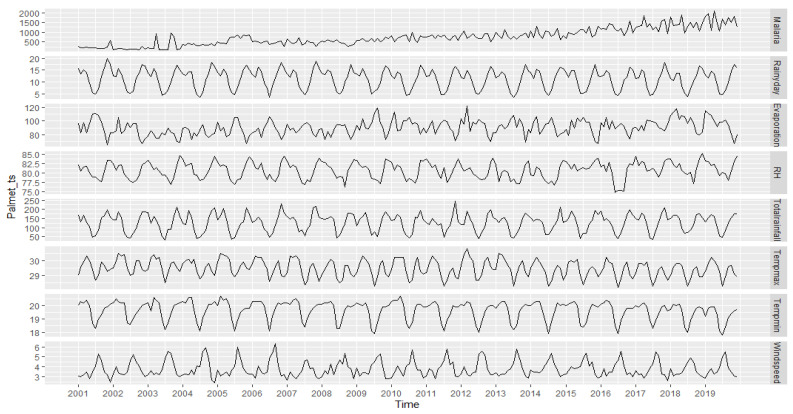
Evolution of study variables from 2001 to 2019. The legends of the variables are shown in the Appendix A and Appendix B. Note: The number of malaria cases is obtained by multiplying the absolute number on the y axis by 10³.

To begin, let us present the temporal evolution of all the variables of the study in the following Charts (Figure 7):

All of the study variables follow an oscillating pattern over the past 19 years. A general trend, probably driven by weather parameters, is detected as well as cyclical trends. Later on, we will see how to predict future occurrences, and the uncertainty of these predictions, from the recorded data.

#### 3.3.1. Identification of the Time Series

**Figure 8:**
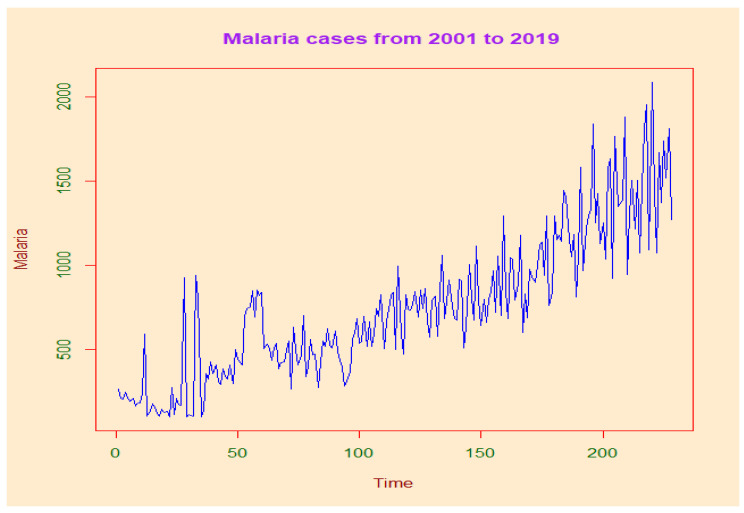
Trend in malaria cases from 2001 to 2019, which illustrate that the evolution of malaria follows an increasing trend with oscillations recorded between 2001 and 2019 in DRC. Note: The number of malaria cases is obtained by multiplying the absolute number on the y axis by 10³.

We use the “Palmet” series, which represents the number of malaria cases between January 2001 and December 2019 in DRC (Figure 8).

An oscillating trend is observed between 2001 and 2004. In contrast, a linear and increasing trend is observed between 2005 and 2019. The variance of malaria cases does not always appear to be constant around the seasonal mean in the short term, but it remains relatively stable over longer periods. The annual cycle is also clearly repetitive. This Chart confirms the sharp increase in malaria episodes in the first and last quarter. The downward trend is observed in the third quarter.

#### 3.3.2. Modeling Choices

**Figure 9:**
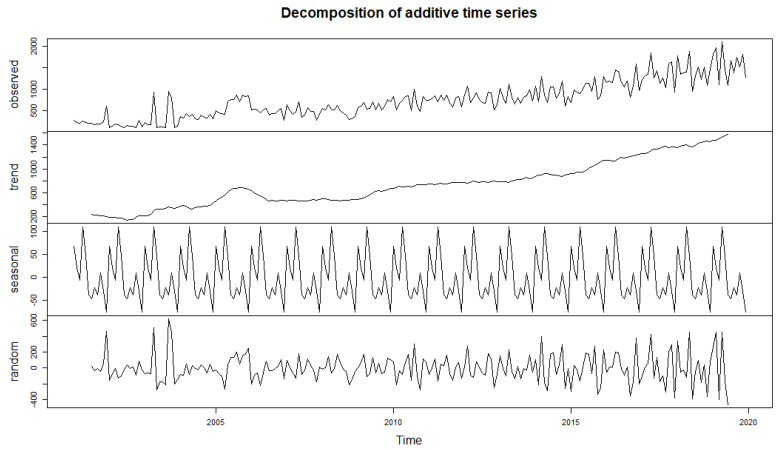
Decomposition of the “Palmet” time series. Note: The numbers on the y-axis represent the absolute number multiplied by 1000.

In order to model the stochastic part of the series, we start by removing the deterministic part (trend + seasonality). We can then identify and model the stochastic part. In this case, we use the moving average method. We estimate the trend and the seasonality in a non-parametric way by the moving average method (Figure 9).

Palmet: Our forecasting model was named weather malaria (in French “paludisme météorologie”).

The “Palmet” series follows a linear trend over the last 19 years. However, there are seasonal fluctuations that oscillate around the general trend on a regular basis during the study period. After decomposing the Palmet series, the random (stochastic) component shows low variabilities around the mean. This justifies a modeling of the Stochastic part.

**Figure 10:**
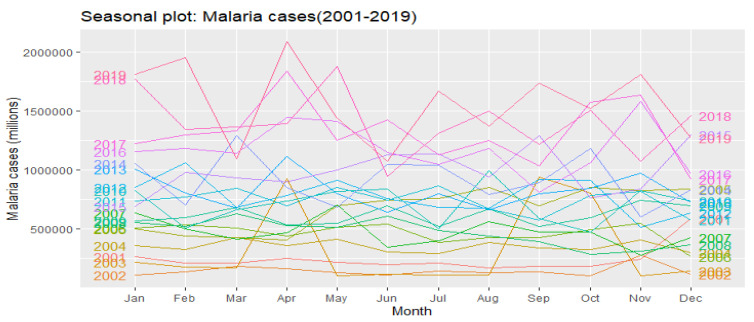
Seasonality of malaria episodes in DRC from 2001 to 2019.

Over the past 19 years, malaria episodes have shown high monthly variability around the average, with a peak in April. It decreases to lowest values in June, but rises again to maximum in July, September, October and November (Figure 10).

#### 3.3.3. Diagnosis of Seasonality

To diagnose seasonality, we used the autocorrelation test. Just as correlation measures the extent of a linear relationship between two variables, autocorrelation measures the linear relationship between lagged values in a time series.

If the test of stationarity of the series were confirmed, by “difference”, the second step in the diagnosis of the series would be the analysis of autocorrelations. Two indicators are then used: Total autocorrelation (ACF) and partial autocorrelation (PACF). These indicators can be examined by Charts or by numerical analysis.

There are several autocorrelation coefficients, corresponding to each panel in the shift plot. For example, *r*_1_ measures the relationship between *y_t_* and *y_t_*_−1_, *r*_2_ measures the relationship between *y_t_* and *y_t_*_−2_ and so on.

The top ten autocorrelation coefficients for the mean annual malaria cases data are shown in the following Table 1:

The Box-Pierce test was used to deduce the significance of total autocorrelations (ACF) at the 5% significance level.

The null hypothesis of these tests is H0: suggests the absence of autocorrelation (between 1 and the maximum lag indicated in parameter). Below is the implementation of the Box-Pierce test in R:

data: Palu

X-squared = 2284.5, df = 24, *p*-value < 2.2 × 10^−16^.

We reject H0 because *p*-value = 2.2 × 10^−16^ << 5%.

The autocorrelation coefficients are plotted to show the autocorrelation function known as a correlogram.

For the total autocorrelation (ACF), we see that *r*_1_ is higher than for the other lags. This is due to the seasonal pattern of the data: peaks tend to be one quarter apart and troughs one quarter apart. All total autocorrelations (ACF) are significant. As for partial autocorrelation (PACF), *r*_6_, *r*_8_, *r*_9_ and *r*_10_ are more negative than for the other lags because the troughs tend to be two quarters behind the peaks.

#### 3.3.4. Charts Visualization: Correlograms

**Figure 11:**
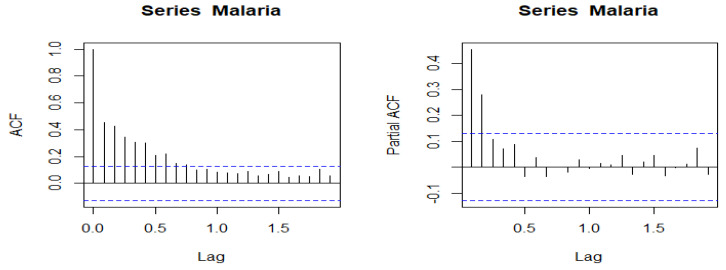
Total and partial autocorrelation. The blue dashed lines indicate whether the correlations are significantly different from zero.

A total autocorrelation (a partial autocorrelation) is significant at a lag of order *p* (resp. order q) when the “bar” at that lag falls outside the confidence interval (except for the lag of order 0, the first bar). It can be seen that for the total autocorrelation (ACF), autocorrelations of order 1 to 10 are significant. For PACF, the significant partial autocorrelations are those of order 1 to 2 (Figure 11).

The performance of the predictions depends on the size of the prediction errors. It is a matter of comparing the predictions with the actual observed values. The smaller the training data, the better the method performs (Table 2).

**Figure 12:**
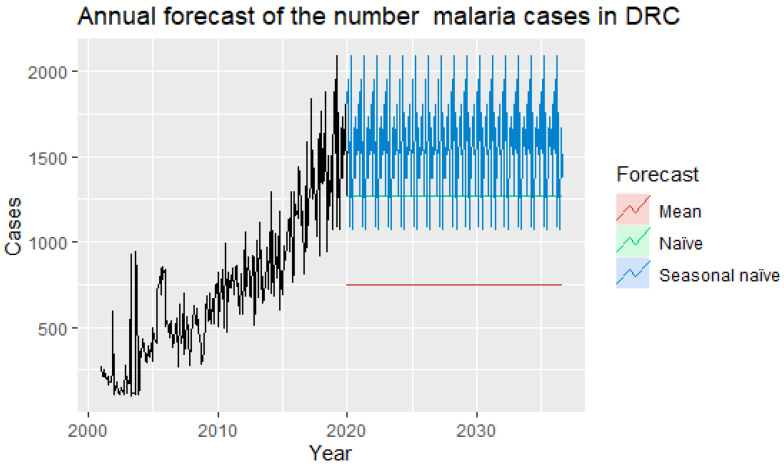
Annual forecast of malaria cases to the end of 2036. Note: Malaria cases are obtained by multiplying the absolute number on the y-axis by 10³.

It is obvious from the Charts below that the naive seasonal method is the best for these data, although it can still be improved, as we will discover later. Sometimes different measures of accuracy lead to different results as to which forecasting method is more appropriate. However, in this case, all results indicate that the seasonal naive method is the best of the three methods for this data set (Figure 12). The results can be seen in Charts below (Table 3).

Here, the best method is the seasonal naive method (regardless of the accuracy measure used).

#### 3.3.5. Forecast Combinations

**Figure 13:**
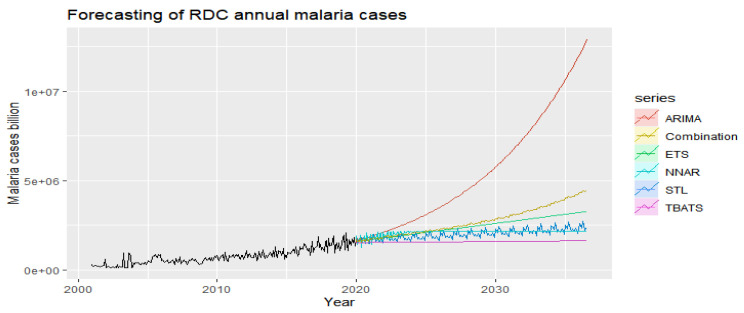
Point forecasts from various applied methods of malaria episodes in DRC.

Using malaria case count data from the last 19 years (228 months) of observations, we modeled and predicted future malaria infestations for the next 200 months using forecasts from the following models: ETS, ARIMA, STL-ETS, NNAR, and TBATS; and we compare the results (Figure 13).

The combined model approach predicts an exponential increase in malaria episodes over the next 16 years in DRC. The combinatorial approach was used to improve the accuracy of the forecasts.

#### 3.3.6. Predictive Models

The NNAR is particularly well suited to this series while the combined approach is twice as good as the component method. In the next 16 years, there will be an average increase of 309,198 new malaria infestations per year per province (Table 3).

#### 3.3.7. Final Model Selection

Before selecting the final model for the prediction, several models are proposed and compared (Table 4).

The time series models presented above allow for the inclusion of information from past observations of a series, but not for the inclusion of other information that might also be relevant. If we want to include external variables that can explain some of the historical variation and can lead to more accurate forecasts, we need to exploit dynamic regression models that take into account the subtle variation in time series. In this case, we will use the deterministic and stochastic model and then compare them.

A deterministic trend is obtained by using the regression model:

$$y_{t}=\beta_{0}+\beta_{1}t+\eta_{t},$$

where η*_t_* is an ARMA process.

The following equations are generated:

$$y_{t}=4414.273+6420.4719t+\eta_{t}$$

$$\eta_{t}=0.9749\eta_{t-1}-0.8733\eta_{t-2}+\varepsilon_{t}$$

$$\varepsilon_{t}∼NID\left(40450000000\right)$$

Each year, there will be an average of 6420 of the new malaria cases per health facility.

Our stochastic trend model is estimated as follows (Table 5):

The model can be written as:

$$\;y_{t}-y_{t-1}=6024.074+{\eta^{\prime }}_{t}$$

$$y_{t}=y_{0}+6024.074t+\eta_{t}$$

$${\;\eta }_{t}=\eta_{t-1}+0.1068\varepsilon_{t-1}+\varepsilon_{t}$$

$${\;\varepsilon }_{t}∼NID\left(38140000000\right)$$

**Figure 14:**
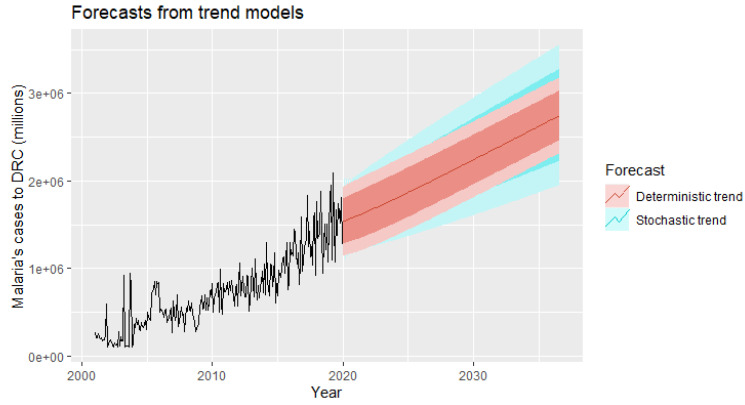
Forecast of the annual number of malaria cases in DRC using a deterministic trend model and a stochastic trend model.

In this case, of the stochastic model, the annual increase in malaria cases is 6024.07 per health facility. Although the growth estimates are similar, the prediction intervals are not, as shown in Figure 14. In particular, the stochastic trends have much wider prediction intervals because the errors are not stationary.

There is an implicit assumption with deterministic trends that the slope of the trend will not change over time. On the other hand, stochastic trends can change, and the estimated growth is only assumed to be the average growth over the historical period, not necessarily the growth rate that will be observed in the future. Therefore, it is safer to forecast with stochastic trends as our final choice of model, especially for longer forecasting horizons, because the forecasting intervals allow for greater uncertainty about future growth.

### 3.4. Climate Predictors Associated with Malaria

We also modelled the relationship between malaria morbidity and climate factors between 2001 and 2019 adjusting for spatio-temporal variations to identify climatic predictors of malaria (Table 6).

Legend:

RD: Rainy day (day/month)

TEUS: Total evaporation under shelter (pitcher)

P24: Precipitation of 24 H (mm)

Tmin: Min. temperature (°C)

WS: Wind speed at 2 m above the ground (m/s)

RH: Relative humidity (%)

**Figure 15:**
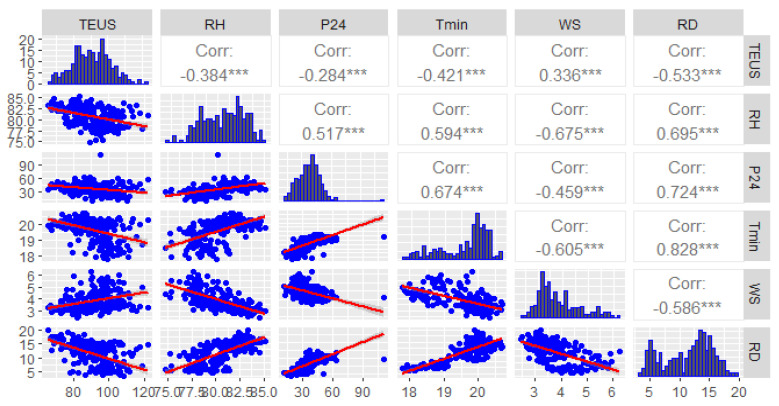
Scatterplot matrix of Spearman correlation. [*** indicates statistically significant *p* < 0.0001].

Figure 15 shows the correlation matrix. Spearman correlation analysis indicates a statistically significant association between evaporation within two meters of the ground and the number of annual malaria cases (r = 0.25; *p* < 0.001).

A statistically insignificant association was observed between the rest of the climate variables and the number of annual malaria cases.

Based on the historical average values of geoclimatic factors (all else being equal), over the next 16 years, the number of malaria cases will increase by its historical average value of 653,071 cases per year.

In addition, for each annual increase of one unit of evaporation at two meters above ground, the absolute number of malaria cases will increase by its historical average value of 13,285 cases. This change is statistically significant (*p* < 0.0001).

Year-over-year, when relative soil moisture increases by an average of one percent, the average number of malaria cases will increase by an average of 39,233 cases. A statistically significant change (*p* = 0.049).

Similarly, the annual increase in heavy rainfall in millimeters will result in an increase in malaria cases from its historical average of 3058 cases per year. This increase is statistically significant (*p* = 0.049).

Similarly, when the density increases by one inhabitant per square kilometer from one year to the next, the average number of malaria cases will increase by 1320 cases compared to its historical value (*p* < 0.001).

On the other hand, a one-degree Celsius increase in minimum temperature each year will result in an average decrease in malaria cases by 23,287 of its historical average value per year (*p* = 0.008).

## 4. Discussion

Malaria is caused by infection with protozoan parasites of the species Plasmodium. Plasmodium falciparum is widespread in Africa, while *P. vivax*, *P. ovale* and *P. malariae* infections are less common and geographically limited [14,15].

Globally, the number of malaria cases is estimated to be 228 million in 2018 (95% confidence interval [CI]: 206–258 million), compared with 251 million in 2010 (95% CI: 231–278 million) and 231 million in 2017 (95% CI: 211–259 million) [16].

In DRC, our results show an average malaria prevalence of the last 19 years of 13,246 (11,783.83–1417.83) cases per 100,000 people at risk. This prevalence increases significantly during the whole study period. The year 2002 was more morbid with 29,137.99 (12,094.51–38,304.56) cases per 100,000 persons at risk.

Most cases in 2018 (213 million or 93%) were recorded in the WHO African region, far ahead of the Southeast Asian region (3.4%) and the Eastern Mediterranean region (2.1%) [16]. Our study showed that malaria-attributable mortality had reached 117.33 (71.08–163.58) malaria deaths per 100,000 people exposed to malaria in 2002. In 2008, the lowest level recorded was 19.04 (14.14–23.35) malaria deaths per 100,000 people exposed to malaria. In general, malaria mortality has been declining dramatically over the past 19 years.

The same observations were made by WHO 2019 showing that on a global scale, malaria incidence declined between 2010 and 2018 from 71 cases per 1000 people at risk of malaria to 57 per 1000. Nevertheless, this decline slowed considerably between 2014 and 2018, with incidence decreasing to 57 per 1000 in 2014 and remaining at a similar level through 2018 [16].

The WHO Africa region alone accounted for 94% of global malaria deaths in 2018. Yet, it also accounted for 85% of the 180,000 fewer deaths from the disease compared to 2010 [16]. Nearly 85% of global malaria deaths in 2018 were concentrated in 20 countries in the WHO African region and India. Nigeria alone accounted for nearly 24% of these deaths, followed by the Democratic Republic of Congo (11%), the United Republic of Tanzania (5%), and Angola, Mozambique, and Niger (4% each) [16].

In our study, Spearman’s correlation analysis indicates a statistically significant association between evaporation at two meters from the ground and the number of annual malaria cases. A statistically insignificant association was observed between the rest of the climate variables and the number of annual malaria cases. In contrast, in South Africa, a statistically significant association between all climate variables studied and monthly malaria cases was reported where minimum temperature had the highest correlation (R = 0.39; *p* < 0.001), followed by total quantity of rainfall at two meters altitude and mean temperature (r = 0.35; *p* < 0.001, r = 0.35; *p* < 0.001), then relative humidity and maximum temperature with (R = 0.29; *p* < 0.001, r = 0.25; *p* < 0.001), respectively [17].

This difference could be related to the disparity in transmission levels in Africa, both in intensity and in seasonality and regularity. This disparity has consequences at several levels: biological, immunological, pathological, etc. Subjected to a strong and permanent plasmodial infection, the population develops a premunition, a temporary immunity, non-sterilizing and maintained by the infection, itself maintained by the transmission.

It should be noted that South Africa is located in an area where transmission is regular every year, with a long seasonal interruption of about 6 months, linked to the rhythm of the rains. The populations of these areas have a very high degree of resistance to malaria but are not totally free of “moderate” malaria attacks and may be susceptible to severe malaria attacks in case of plasmodial infections by foreign strains [18].

Wilson’s classifications of epidemiological facies as “models” reflect the dynamics of host-vector-parasite relationships in different environments, and he points out that all intermediate situations can be found within and between these basic models.

There are seasonal variations in the intensity of transmission, but no interruption, however brief. Transmission is generally by An. gambiae and An. funestus. This mode of transmission is well illustrated by the degraded forest areas of Central Africa (DRC, Congo, Cameroon, etc.).

This approach by Wilson highlights the transmission/malaria disease relationships, taking into consideration the intensity of transmission (chance of infection or frequency of infection), the duration of transmission with its regularity from one year to the next (transmission continues nearly all-year round or annually recurring season or seasons of malaria transmission), the ecological variables (altitude, temperature, humidity) with the relative development of immunity. Variations in these different variables are reflected in variations in the classical malaria indices (splenic index, plasmodic index) but above all in malaria morbidity according to age groups and general mortality in the event of an epidemic.

Some authors agree with Wilson’s categorization of epidemiological facies where the DRC/Zaire is located in the Degraded Forest Zone: permanent transmission (=Wilson’s Group I): where malaria is endemic with intense and permanent transmission [19].

The results of our study reveal a negative but not statistically significant association observed between the remaining number of annual malaria cases and wind speed, number of rainy days, maximum and minimum temperature. Our results concur with those found by Lindsay et al. showing a negative but statistically significant association between rainfall and malaria cases, which could be due to the fact that the breeding sites were washed away by heavy rains [20].

The significant relationships observed with a lag of more than one month between malaria onset and associated climatic variables (*p* < 0.05), such as rainfall and minimum temperature at two months, suggest that climatic conditions in a given year (early onset of rains, especially those associated with La Niña conditions and tropical cyclones) may affect malaria transmission in the following year.

Periods of heavy rainfall combined with low temperatures and high relative humidity promote saturated soil moisture, which prolongs the life of water pockets and may result in the persistence of larval habitats [21,22].

Several forecasting models were used in this study to predict future malaria episodes. Some authors used the SARIMAX model to forecast monthly time series of malaria cases using the SARIMA Box-Jenkins approach and multiple linear regression (MLR). The forecast shows that the number of malaria cases remains high until July, which is normally a period of low malaria transmission, indicating a change in the malaria season [17].

For Kumar et al. [23], the autoregressive integrated moving average, ARIMA (0,1,1) (0,1,0) 12, was the best fitting model and could explain 72.5% variability in the time series data. Rainfall (*p* = 0.004) and relative humidity (*p* = 0.001) were found to be significant predictors for malaria transmission in the study area. The seasonal adjustment factor (SAF) for malaria cases showed a peak during August and September. Although ARIMA time series analysis models are a simple and reliable tool for producing reliable forecasts for malaria in Delhi, India, in our study however, the combination of several forecasts led to higher forecast accuracy.

The combination of multiple model forecasts (ETS, ARIMA, STL-ETS, NNAR, and BATS) fit better and led to greater accuracy in modeling and forecasting for future malaria infestations over the next 200 months. The results were virtually unanimous: the combined model approach predicts an average increase of 309,198 new malaria infestations per year per province over the next 16 years in DRC. However, the NNAR predictive model was particularly well suited in this series.

Wangdi et al. [24] also found that ARIMA (2,1,1) (0,1,1) 12 was the best possible model for predicting malaria cases in Bhutan. In the same vein, The ARIMA model has also been used for malaria case prediction in Sri Lanka [25] and Ethiopia [26]. To evaluate the predictive performance of our model, we divided our data set into a training set and a test set of sizes 90% and 10%, respectively.

The mean absolute scaled error (MASE) as a measure of the quality of the predictions made by our models showed that the naive seasonal method was the best of the three methods for this data set with a MASE of 0.894. Recall that the closer the MASE is to zero, the better the forecast.

Our forecasting horizon was set at 2036, and it was desirable and wise to finally retain the stochastic model, as it is safer to forecast with stochastic trends, especially for longer forecasting horizons, as the forecasting intervals allow for greater uncertainty about future growth.

Our stochastic model predicts an average annual increase of 6024.07 new malaria cases per facility.

As for the estimated seasonal component, this study shows a slight decrease in malaria cases from January to February throughout the study period, rising again in March and reaching the peak in April. It declines to lowest values in June, but rises again to the peak in July, September and November. In addition, malaria is present almost year-round in the DRC due to the tropical climate and the country’s extensive river system. The DRC has about 30 major rivers with at least 20,000 km of shoreline and 15 lakes with about 180,000 km of shoreline.

As for the “seasonality” component, this study reveals that the “Palmet” series will have variation between months simply due to the different number of days in each month, in addition to the seasonal variation in future years. This disparity is closely related to the variability of precipitation during the study period showing very intense palustrine episodes from January to June and then from September to December. On the other hand, a decrease in rainfall is observed between July and October (dry season).

The relief of the DRC can also have an impact on the seasonality of malaria. There is a vast low-lying area in the center of the country, which is a plateau shaped by the westward flowing river basin and covered by a large tropical forest. This area is surrounded by mountainous terraces such as the Mitumba Mountains in the east and the Virunga Mountains in the north, savanna-covered plateaus in the south and southwest, and the north is bordered beyond the river by dense forest. High mountains are found at the eastern end of the country (Great Rift region).

A study conducted in Kenya shows an increase in malaria cases in the high and medium altitude areas while in the riverine and lowland areas, the number of malaria cases had significantly decreased as of 2011, during the study period [27].

Finally, our results of the multivariate time series analysis showed that over the next 16 years, the number of malaria cases will increase from its historical average value of 653,071 cases per year due to increase in geo-climatic factors such as evaporation under shelter at two meters from the ground, relative soil moisture, high rainfall and population growth with a density of one inhabitant per square kilometer will significantly increase the number of malaria cases from its historical mean value each year. On the other hand, increasing the minimum temperature by one degree Celsius each year will lead to an average decrease in malaria cases by 23.287 of its historical average value per year.

Our previous study conducted in 2022 showed that the number of rainy days resulted in a statistically significant 7% increase in malaria cases with malaria morbidity significantly associated with geographic location, total evaporation under shelter, maximum daily temperature at two meters altitude, and humidity at two meters altitude [28].

Our results are similar to those of Solomon Kibret et al. in their study on environmental and meteorological factors related to malaria transmission around large dams in three ecological settings in Ethiopia, which showed that Rainfall (lagged 1 and 2 months) was significantly associated with malaria incidence, but only at the lowland dam, while minimum and maximum air temperatures (lagged 1 and 2 months) were significant factors only at the highland dam [29].

The polynomial model Mopuri R et al. had predicted malaria cases with high predictive power and revealed that first lag malaria cases and population play a vital role in malaria transmission. Similarly, mean temperature, rainfall and normalized vegetation index had significant impact on malaria cases [30].

In the same perspective, the results of Nkiruka et al. suggest that, although the exact association between malaria incidence and climate variability varies across geographic regions, non-seasonal changes in three climatic factors (rainfall, temperature, and surface radiation) contribute significantly to malaria occurrence [31].

The results of Sahai et al. agree with ours. The authors note that changes in rainfall and temperature regimes probably play a major role in increasing the incidence of these diseases in different geographical locations. A detailed analysis was conducted on the incidence of malaria and diarrhea in two districts of Maharashtra State. It is found that the increased likelihood of high (low) rainfall, high (low) minimum temperatures and low (moderate) maximum temperatures are more (less) conducive to both diseases in these areas, but with different thresholds [32].

## 5. Conclusions

The purpose of this study was to forecast malaria episodes to 2036 using the epidemiological and meteorological model in order to strengthen the surveillance and response to diseases with epidemic potential throughout the DRC. The results of the predictive analysis showed that malaria transmission is highly seasonal in the DRC despite being endemic in the country.

Our stochastic trend model predicts an average annual increase of 6024.07 malaria cases per health facility. As such, this model forecasts an exponential growth of epidemic waves over the next 200 months of the study.

Furthermore, the geo-climatic predictors most associated with malaria were geographic location (western, central and northeastern region of the country), total evaporation under shelter, maximum daily temperature at two meters altitude. Finally, the average number of malaria cases increased positively as a function of the average number of rainy days, the total quantity of rainfall and the average daily temperature.

Therefore, the results of this research should provide relevant information for the Congolese government to respond to malaria in real time by setting up a warning system integrating the monitoring of rainfall and temperature trends and early detection of anomalies in weather patterns.

## Figures and Tables

**Table 1:** Autocorrelation coefficients.

Coefficient	$r_{1}$	$r_{2}$	$r_{3}$	$r_{4}$	$r_{5}$	$r_{6}$	$r_{7}$	$r_{8}$	$r_{9}$	$r_{10}$
ACF	1.000	0.455	0.428	0.346	0.308	0.301	0.209	0.219	0.146	0.139
PACF	0.455	0.279	0.107	0.071	0.089	−0.037	0.038	−0.037	−0.001	−0.020

**Table 2:** Test for evaluating the accuracy of the forecasts.

Coefficient	Test	RMSE	MAE	MAPE	MASE
Mean method	Training set	282,904.4	214,217.7	34.25161	1.0000000
	Test set	188,203.0	188,203.0	14.82961	0.8785594
Naïve method	Training set	430,145.4	341,277.7	89.74833	1.593135
	Test set	524,529.7	524,529.7	41.33075	2.448582
Seasonal naïve method	Training set	276,096.8	191,684.1	33.11892	0.8948098
	Test set	541,065.0	541,065.0	42.63366	2.5257712

**Table 3:** Predictive models for malaria episodes over the next 16 years.

Model	ETS	ARIMA	STL-ETS	NNAR	TBATS	Combination
Value	341,546.2	503,800.4	214,444.0	151,692.0	334,506.0	309,197.7

**Table 4:** Deterministic model (Regression with ARIMA (1,0,1).

	Ar1	ma1	xreg
errors Coefficients	0.9749	−0.8733	6420.4719
se	0.0206	0.0419	419.2883
$\sigma^{2}$: 4.045 × 10^10^			
log likelihood: −3106.59			
AIC: 6221.19			
BIC: 6234.9			

**Table 5:** Stochastic model (ARIMA (2,1,2).

	AR1A	AR2	MA1	MA2	DRIFT
Coefficients	−1.0052	−0.1915	0.1068	−0.8216	6024.074
se	0.0763	0.0758	0.0477	0.0471	1711.044
σ^2^: 3.814 × 10^10^					
log likelihood = −3086.09					
AIC: 6184.18					
BIC = 6204.73					

**Table 6:** Multivariate time series analysis of Geoclimatic factors and Malaria Morbidity.

Explanatory Variables	β	IC 95%	Pr (>|F-Statistic|)
(Intercept)	653,071	−4,549,211 to 5,855,353	0.804
Evaporation	13,285	7632 to 18,937	0.00001
Soil moisture	39,233	28 to 78,438	0.049
Total rainfall quantity	3058	198 to 5919	0.036
Minimun temperature	−184,707	−322,025 to −47,390	0.008
Wind speed	−8833	−104,307 to 86,641	0.855
Precipitation 24 h	4581	−2428 to 11,590	0.1991
Average number of rainy days	−23,287	−61,872 to 15,297	0.235
Density	1320.87	(1114.22 to 1527.51)	<0.001

## Data Availability

We exploited the databases of the Directorate of Epidemiological Surveillance and the Mettelsat database for meteorological data centralizing data from 26 provincial health directorates of the DRC from 2001 to 2019. Climatic data including total rainfall quantity, temperature, wind speed, total evaporation under shelter (piche), average relative humidity, were measured permanently by means of different meteorological stations in the DRC.

## Acronyms

ARIMA	Auto-Regressive Integrated Moving Average
Density	Population density (Population/Km^2^)
ETS	Error Trend and Seasonality
Facies	Facies
Humidity	Average relative humidity (%)
Malariacase	Malaria cases
MaxTemp	Maximal temperature in °C
MinTemp	Minimal temperature in °C
NID	stands for “normally and independently distributed
NNAR	neural network autoregression model
Precipitation24H	total rainfall quantity at 24 h
Rainyday	number of Rainy Days
STL	Seasonal and Trend decomposition using Loess
Totalrainfall	total rainfall (mm)
Windspeed	wind speed (Km/h)
Equat	Equateur
HKAT	Haut Katanga
HL	Haut LOMAMI
KAS	Kasaï
KC	Kasaï Central
KIN	Kinshasa
KO	Kasaï oriental
KGC	Kongo central
LOM	Lomami
MAN	Maniema
SK	Sud Kivu
SN	Nord Ubangi
SU	Sud Ubangi

## Appendix A

**Table A1:** Malaria prevalence rate (P.100.000 persons at risk) in DRC, 2001–2019.

Year	*n*			Prevalence	95% Confidence Interval	
		Total Cases	Person Years at Risk		Lower Bound	Upper Bound
2001	26	2,931,016	57,536,316	5094.20	2918.33	7181.91
2002	26	17,318,171	59,435,015	**29,137.99**	**12,094.51**	**38,304.56**
2003	26	15,021,325	61,396,371	**24,466.14**	**12,109.71**	**38,872.55**
2004	26	4,251,830	63,422,448	6703.98	4406.78	8317.09
2005	26	8,041,313	65,515,392	12,273.93	7332.18	14,726.80
2006	26	5,581,162	67,677,401	8246.71	5065.45	10,856.70
2007	26	5,662,730	69,910,755	8099.94	5248.46	9886.56
2008	26	5,632,064	72,217,808	7798.72	5543.42	9439.33
2009	26	7,372,005	74,600,994	9881.91	7073.72	11,716.57
2010	26	8,515,264	77,062,827	11,049.77	7853.73	12,936.89
2011	26	8,930,122	79,605,900	11,217.91	8249.03	13,792.51
2012	26	9,377,143	82,232,897	11,403.15	9164.74	13,859.17
2013	26	9,842,772	84,946,581	11,587.01	9419.97	13,568.57
2014	26	10,964,357	87,749,819	12,495.02	10,555.09	15,394.83
2015	26	11,890,907	90,645,561	13,118.02	10,669.10	16,135.94
2016	26	14,146,386	93,636,868	15,107.71	13,125.69	18,196.94
2017	26	15,914,057	96,726,882	16,452.57	13,168.19	22,394.50
2018	26	16,760,860	99,816,895	16,791.61	14,362.18	20,381.75
2019	26	18,836,175	103,005,624	**18,286.55**	**15,794.49**	**23,096.55**
Total	494	196,989,659	1,487,142,354	13,246.19	11,783.83	14,174.83

Note: Figures in bold indicate malaria prevalence (P.100,000 persons at risk) significantly higher than the mean for a period (*p* < 0.0001).

**Table A2:** Malaria case-fatality rate (Deaths per 100 cases) in DRC, 2001–2019.

Year	*n*	Lethality	95% Confidence Interval	
			Lower Bound	Lower Bound
2001	26	**0.67**	**0.43**	**0.91**
2002	26	**0.68**	**0.53**	**0.83**
2003	26	0.52	0.38	0.67
2004	26	0.45	0.34	0.56
2005	26	0.33	0.25	0.42
2006	26	0.33	0.26	0.40
2007	26	0.33	0.26	0.39
2008	26	0.32	0.25	0.40
2009	26	0.28	0.22	0.34
2010	26	0.25	0.17	0.32
2011	26	0.29	0.21	0.37
2012	26	0.27	0.19	0.36
2013	26	0.29	0.19	0.40
2014	26	0.27	0.20	0.34
2015	26	0.23	0.18	0.29
2016	26	0.20	0.16	0.24
2017	26	0.15	0.11	0.18
2018	26	0.12	0.09	0.15
2019	26	0.11	0.09	0.14
Total	494	0.32	0.30	0.35

Note: Bolded numbers indicate malaria case-fatality rates that are significantly higher than the average for a given period (*p* < 0.0001). Malaria case-fatality in DRC has been decreasing over the past 19 years, from 0.67 (0.43–0.91) deaths per 100 positive test cases in 2001 to 0.11 (0.09–0.14) deaths per 100 positive test cases in 2019. This case fatality varied significantly throughout the study period (*p* < 0.0001). From 2001 to 2002, malaria caused more deaths in families with a case-fatality rate higher than 0.66%.
